# Three-dimensional simulations of mixed maneuver for three semicircular canalithiasis on the same side

**DOI:** 10.3389/fmed.2026.1782263

**Published:** 2026-03-13

**Authors:** Zhuohao Li, Yanping Yu, Hongsong Dong, Guohui Nie

**Affiliations:** 1 Shenzhen Second People’s Hospital, Clinical Medicine College of Anhui Medical University, Shenzhen, Guangdong, China; 2 Department of Otolaryngology Head and Neck Surgery, The Second People’s Hospital of Shenzhen, The First Affiliated Hospital of Shenzhen University, Shenzhen, Guangdong, China; 3 Shenzhen Clinical Medical Research Center for Otolaryngology Diseases, The Second People’s Hospital of Shenzhen, The First Affiliated Hospital of Shenzhen University, Shenzhen, Guangdong, China

**Keywords:** benign paroxysmal positional vertigo, canalith repositioning maneuver, canalithiasis, servo motor, three-dimensional simulations

## Abstract

**Background:**

The canalith repositioning maneuver (CRM) is an effective method for treating benign paroxysmal positional vertigo (BPPV). Classical CRM is designed mainly for a single semicircular canal (SC).

**Methods:**

A mixed maneuver (MM) hybridized by classical maneuvers was proposed for otoconia of three SCs on the same side. Simulations of otoconia movement induced by classical CRMs and MM were performed on a 3-axis servo motors platform (SMP) with an iPhone containing the aVOR app. MM was performed on BPPV VIEWER to show the otoconia trajectory.

**Results:**

The Epley and Barbecue maneuver were effective for the otoconia of posterior semicircular canal (PSC) and horizontal semicircular canal (HSC), respectively. Similarly, the supine roll test + Gufoni maneuver was effective for otoconia of HSC, and the Dix-Hallpike test + Yacovino maneuver was effective for otoconia of ASC. Furthermore, the MM was effective for free-floating otoconia in HSC, ASC and long arm of PSC on the same side.

**Conclusion:**

The SMP-aVOR model and BPPV VIEWER provide methods for studying otoconia repositioning. The MM showed potential capability to reposition the common types of otoconia in the three SCs on the same side.

## 1 Introduction

Benign paroxysmal positional vertigo (BPPV) is the most common cause of vertigo (1). Based on the underlying mechanisms, BPPV can be classified as canalithiasis (2) and cupulolithiasis (3). Canalithiasis is the most common condition in patients with BPPV. BPPV can affect all three semicircular canals (SCs) on each side: the posterior semicircular canal (PSC), horizontal semicircular canal (HSC), and anterior semicircular canal (ASC). The PSC is the most commonly involved (4), whereas the anterior canal BPPV is rare (5). BPPV is diagnosed using positional tests, such as the Dix-Hallpike test (DHT), supine roll test (SRT) and deep head hanging test, which can alter canaliths (otoconia) location and cause positional nystagmus (6). These tests are performed by clinicians or on mechanical chairs (7). In some cases, sequential positional tests can redistribute otoconia and alter nystagmus patterns, which complicate diagnosis (8). A questionnaire has also been developed for self-diagnosis (9). Canalith repositioning maneuvers (CRMs) have been developed for BPPV treatment and can be performed manually or using machines (10, 11). Classical CRMs include Epley (12), Barbecue (13), Gufoni (14), and Yacovino maneuver (15). CRMs aim to take the head to angles and positions allowing the displaced debris to move out of the affected canal under the influence of gravity. CRM is selected according to affected canal, position of displaced debris within the canal and patient’s physical condition (16). Classical CRMs are designed to treat a single semicircular canal. For patients with multiple otoconia, successive CRMs should be used according to the affected canals (17).

aVOR is an iPhone application (18). The positional change of the otoconia can be simulated by reading the parameters of the mobile phone gyroscope when the head position changes. In this study, using a servo motors platform (SMP) and an aVOR APP, three-dimensional simulations of classical CRMs were conducted, and a mixed maneuver (MM) hybridized by classical CRMs was elaborated as an alternative treatment for three semicircular canalithiasis on the same side. BPPV VIEWER is a downloadable 3D BPPV model controlled by keyboard and mouse in Mac and PC environments (19), we performed MM on BPPV VIEWER to show the movement trajectory of otoconia.

## 2 Materials and methods

### 2.1 Materials

The aVOR APP (available at: https://itunes.apple.com/us/app/avor/id497245573?mt=8) was installed on an iPhone5 (Apple, Inc., Cupertino, CA, United States), and otoconia were set up in the six semicircular canals.

**Figure 1:**
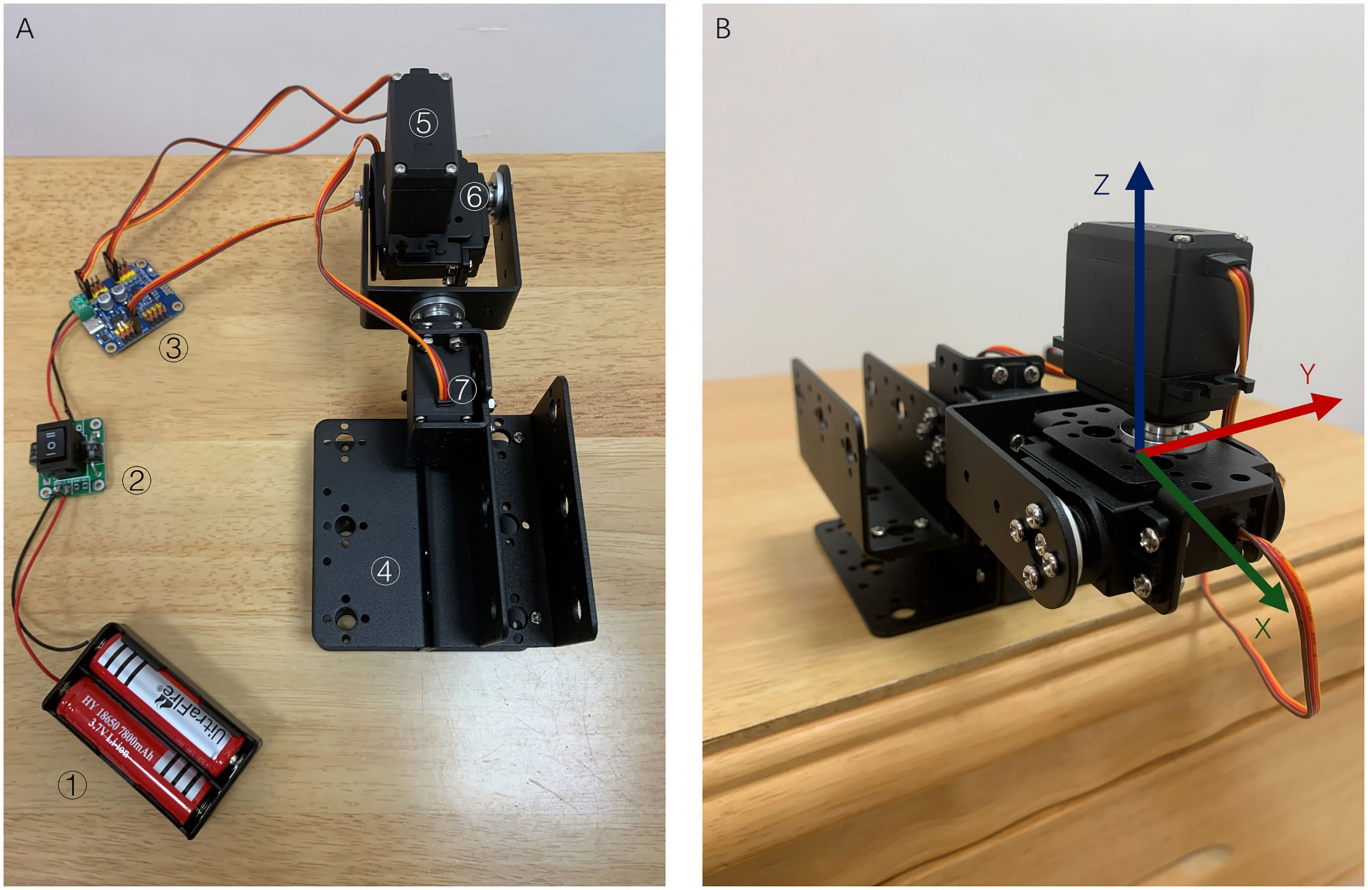
Structure of the SMP and its rotation axes. **(A)** Structure of SMP ① 7.4 V lithium battery; ② Switch; ③ Wit-motion controller; ④ Platform bracket; ⑤ Servo motor z; ⑥ Servo motor y; and ⑦ Servo motor x. **(B)** Rotation axes of the SMP. SMP: Servo motors platform.

The servo motors platform (SMP) (Figure 1) comprises three 180-degree servo motors (SM) and a bracket (WitMotion Shenzhen Co., Ltd., Guangdong, China). The SMs were controlled using a WitMotion controller (WitMotion Shenzhen Co., Ltd., Guangdong, China). Power was supplied by a 7.4 v lithium battery (WitMotion Shenzhen Co., Ltd., Guangdong, China). The rotation directions of the three SM represent the rotation directions of the X, Y and Z axes and follow the right-hand rule.

BPPV VIEWER (available at: https://bppvviewer.com/download/).

### 2.2 Methods

#### 2.2.1 Simulations of CRMs on SMP-aVOR model

CRMs for the right canal were used, SRT and DHT were only performed toward the right side. We waited for 10 s between two successive movements. CRMs were performed as the following description.

Epley maneuver: Step 1, sitting position; Step 2, the head was turned 45° to the right (SM z-45); Step 3, lie back and lean back at 120° (SM y + 120); Step 4, the head was turned 90° left (SM z + 90); Step 5, the head was turned another 90° left (SM x + 90); and Step 6, sitting position (the same SM angle was set as in step 1) (Supplementary Video 1).

Barbecue maneuver: Step 1, sitting position; Step 2, lying down (SM y + 90); Step 3, turned 90° right (SM x-90); Step 4, turned 90° left (SM x + 90); Step 5, turned 90° left (SM z + 90); Step 6, turned 90° left (SM z + 90); Step 7 turned 90° left (SM x + 90); and Step 8, sitting position (SM z-90, SM y-90, SM x-90) (Supplementary Video 2).

Gufoni maneuver: Step 1, sitting position; Step 2, lying on the left side at 90°(SM x + 90); Step 3, the head was turned left at 45° (SM z + 45); and Step 4, sitting position (SM x-90) (Supplementary Video 3).

SRT + Gufoni maneuver: Step 1, sitting position; Step 2, lying down (SM y + 90); Step 3, turned 90° right (SM x-90); Step 4, turned 90° left (SM x + 90); Step 5, sitting position (SM y-90); Step 6, lying on the left side at 90° (SM x + 90); Step 7, the head was turned 45° left 45 (SM z + 45); and Step 8, sitting position (SM x-90) (Supplementary Video 4).

Yacovino maneuver: Step 1, sitting position; Step 2, lying back and leaning back at 120° (SM y + 120); Step 3, the head was lifted at 60° (SM y-60); and Step 4, sitting position (SM y-60) (Supplementary Video 5).

DHT + Yacovino maneuver: Step 1, sitting position; Step 2, the head was turned right at 45° (SM z-45); Step 3, lying and leaning back at 120° (SM y + 120); Step 4, sitting position as in Step 1 (SM y-120, SM z + 45); Step 5, lying and leaning back at 120° (SM y + 120); Step 6, the head was lifted at 60° (SM y-60); and Step 7, sitting position (SM y-60) (Supplementary Video 6).

**Figure 2:**
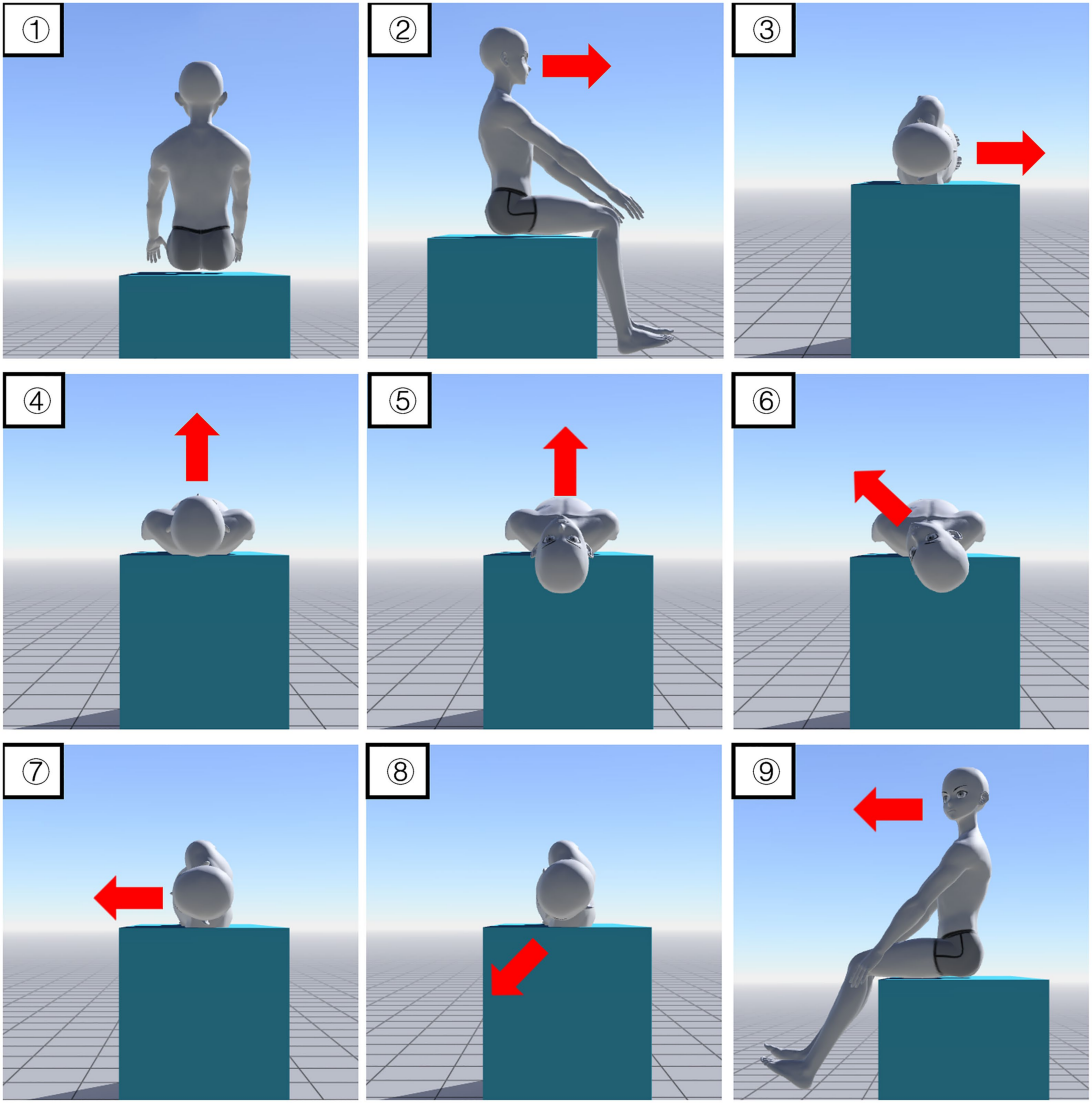
Mixed maneuver. ① Sit on the bed facing the end of the bed; ② Turn 90°; ③ Lie on the right side at 90°; ④ Turn left 90° and lie flat; ⑤ Lean back at 30°; ⑥ Turn 45° left; ⑦ Lie on the left side at 90°; ⑧ Turn 45° left; ⑨ Sit-up (45-degree head-down). Red arrows indicate head direction.

Mixed maneuver: Step 1, sitting position; Step 2, turning 90° right (SM z-90); Step 3, lying on the right side 90° as in Step 3 of Barbecue maneuver (SM y + 90); Step 4, turning 90° left as in Step 4 of Barbecue maneuver (SM z + 90); Step 5, the head was leaned back at 30° as in Step 2 of Yacovino maneuver (SM y + 30); Step 6, the head was turned 45° left as in Step 4 of Epley maneuver (SM z + 45); Step 7, lying on the left side at 90° as in Step 2 of Gufoni maneuver (SM z + 45, SM y-30); Step 8, the head was turned 45° left as in Step 3 of Gufoni maneuver (SM x + 45); and Step 9, sitting position (set the same SM angle as in Step 1) (Supplementary Video 7; Figure 2).

#### 2.2.2 Simulation of MM on BPPV VIEWER

**Figure 3:**
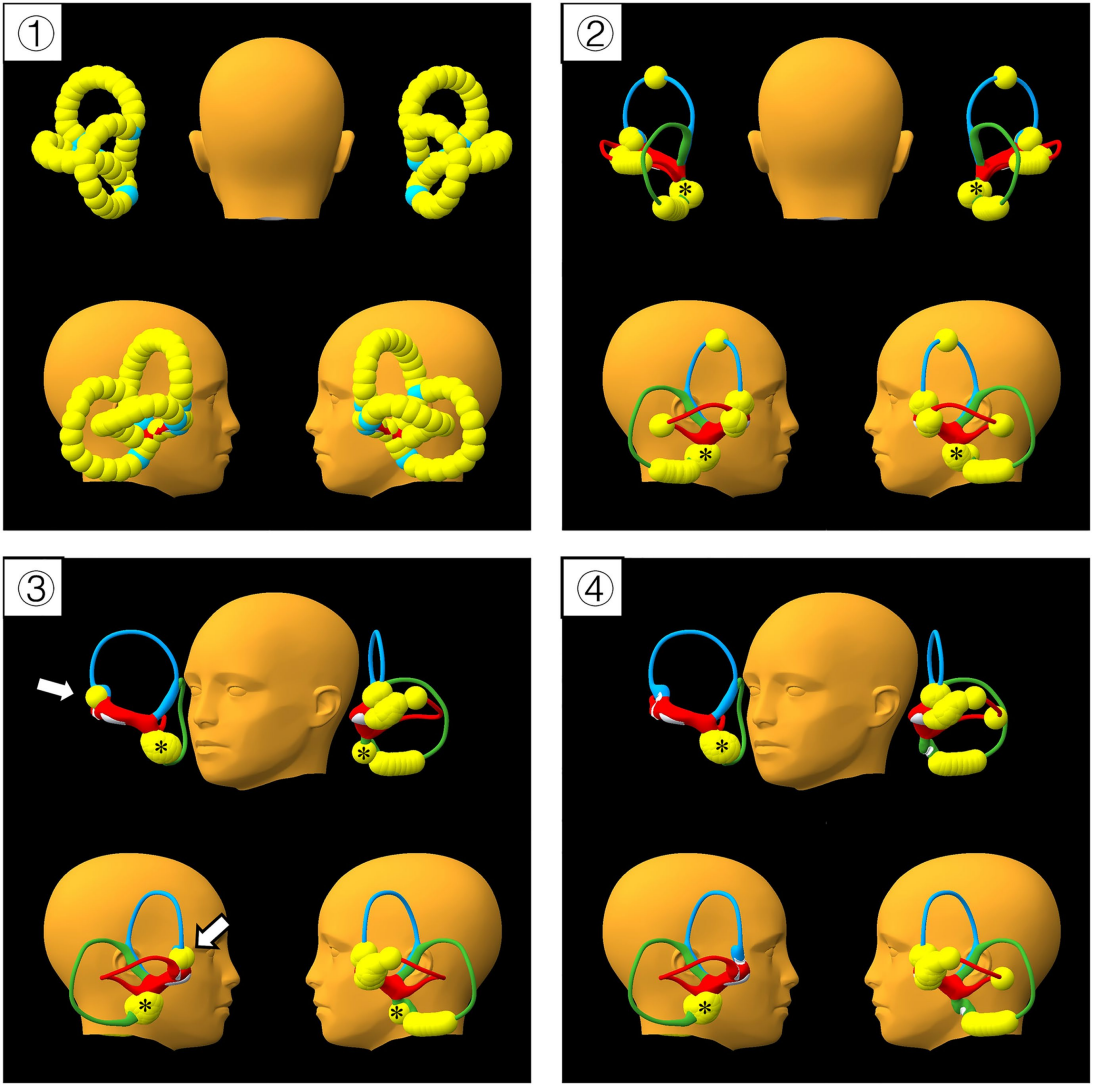
High canalith burden test of the mixed maneuver. ① Successive otoconia were set up before gravity activated; ② Step 1 of MM, most of the otoconia located in the ampulla and its surroundings ③ The distribution of otoconia after one procedure of MM; ④ The distribution of otoconia after 2 procedures of MM. Yellow spheres represent otoconia; blue, green, and red cannals stand for the anterior (ASC), posterior (PSC), and horizontal semicircular canals (HSC), respectively. White arrows indicate the otoconia trapped in the ampulla of ASC, * indicate the otoconia in the utricle.

High canalith burden test: otoconia were placed in succession on all SCs at the maximum load capacity of the model (Figure 3). Right side MM was then performed twice as described above.

**Figure 4:**
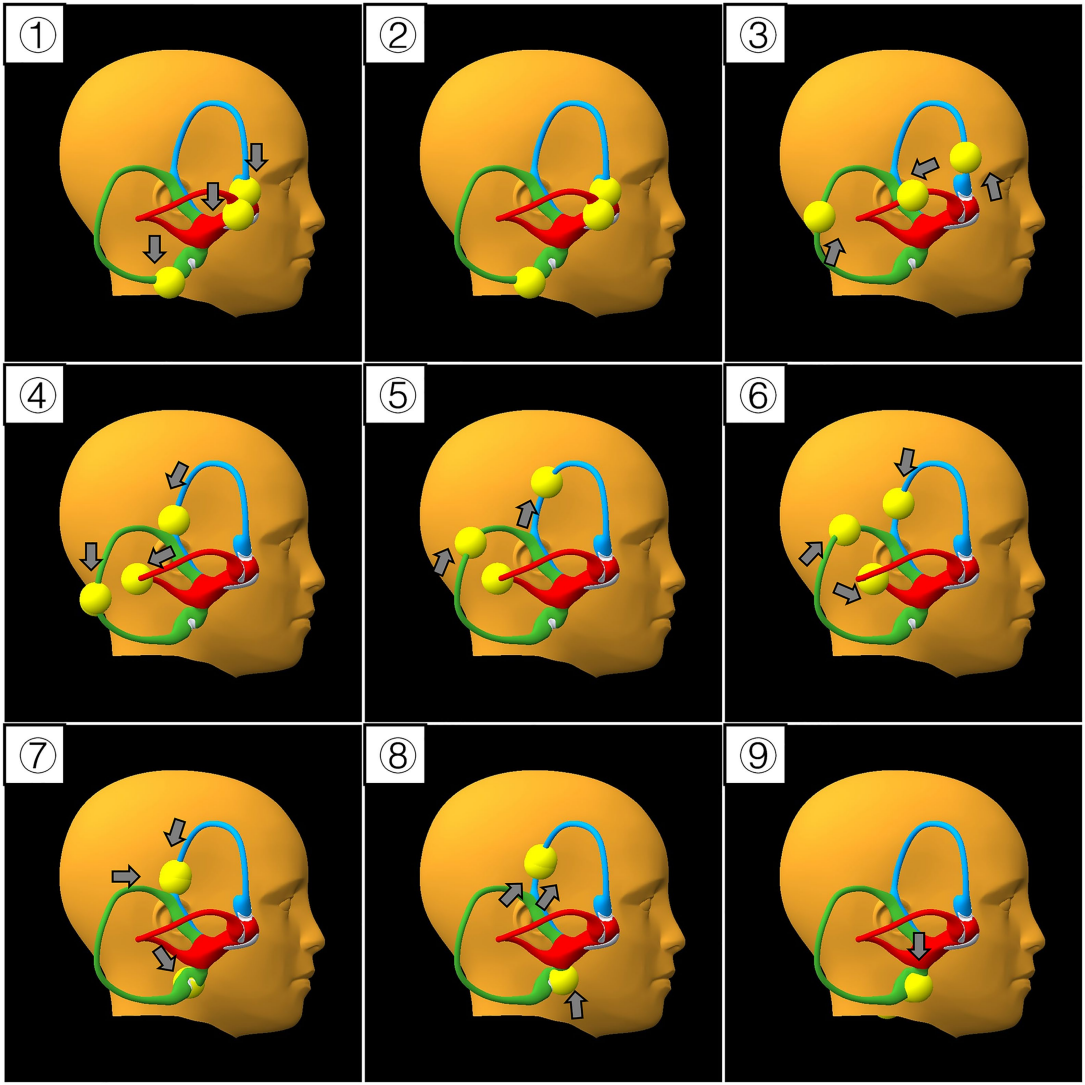
Movement trajectory of otoconia during the mixed maneuver. (①–⑨) Denote step 1 to 9 of the mixed maneuver, respectively. Yellow spheres represent otoconia; blue, green, and red canals stand for the anterior (ASC), posterior (PSC), and horizontal semicircular canals (HSC), respectively. Grey arrows indicate the direction of otoconia movement.

Movement trajectory of otoconia: otoconia were placed in the ampulla on the right side SCs. Right side MM was performed once as described above. We observe the otoconia movement from the right perspective view (Figure 4).

## 3 Results

### 3.1 Simulations of CRMs on SMP-aVOR model

Different CRMs for the right canals were used to observe the effects on otoconia of the six SCs (Supplementary Table 1). The initial state was the sitting position. In this position, the otoconia of HSC and ASC were located in the ampullary arm and trapped in the ampulla, the otoconia of PSC were at the lowest point of the long arm.

#### 3.1.1 Classical CRMs

Epley maneuver: In Step 1and Step 2, otoconia were in initial position without stimulation; In Step 3, the otoconia of SCs on the right side moved toward the utricle; In Step 4 and Step 5, except the otoconia of right HSC, otoconia moved further toward the utricle; In Step 6, the otoconia of PSC and ASC on the right side kept moving toward the utricle while no movement occurred on the otoconia of PSC and HSC on the left side, the others moved toward the ampulla; In Step 7, only otoconia of right PSC entered the common crus and went back into the utricle (Supplementary Video 1).

Barbecue maneuver: In Step 2, the otoconia of PSC in both sides moved toward the utricle; In Step 3, the otoconia of HSC and ASC on the right side moved further toward the utricle; From Step 4 to Step 8, only the otoconia of right HSC kept moving toward the utricle and finally went back into the utricle, the others moved toward the ampulla or remained stationary from Step 6 to Step 8. The otoconia on the left HSC remained in the ampullary arm (Supplementary Video 2).

Gufoni maneuver: From Step 1 to Step 4, no movement occurred on the otoconia of HSC and ASC on the right side which were trapped in the ampulla. In Step 2, otoconia of SCs on the left side moved toward the utricle; In Step 3, all otoconia remained stationary. In Step 4, No otoconia were cleared (Supplementary Video 3).

SRT + Gufoni maneuver: In Step 2, the otoconia of PSC in both sides moved toward the utricle; In Step 3, the otoconia of HSC and ASC on the right side moved away from the ampulla and moved toward the utricle; In Step 4, the otoconia of right HSC kept moving toward the utricle and entered the nonampullary arm; In Step 5, because of the sitting position, the otoconia of PSC in both sides and right anterior canal moved toward the ampulla; In Step 6, the otoconia of right HSC kept moving toward the utricle, the others’ movement was the same as that in Step 2 of Gufoni maneuver; In Step 7, the otoconia of right HSC kept moving toward the utricle and finally went back into the utricle while the otoconia of left HSC moved toward the ampulla; In Step 8, the otoconia of right HSC were cleared. Right-sided SRT promoted the otoconia of the right HSC into the nonampullary arm, and then Gufoni maneuver cleared the nonampullary arm otoconia of the right HSC (Supplementary Video 4).

Yacovino maneuver: In Step 2, the otoconia of PSC in both sides moved toward the utricle; In Step 3 and Step 4, the otoconia of PSC in both sides moved toward the ampulla; From Step 1 to Step 4, the otoconia of the HSC and ASC were trapped in the ampulla, no movement occurred (Supplementary Video 5).

DHT + Yacovino maneuver: In Step 1and Step 2, otoconia were in initial position without stimulation; In Step 3, otoconia of SCs on the right side moved toward the utricle; In Step 4, the otoconia of PSC and ASC on the right side moved toward the ampulla; From Step 5 to Step 7, the otoconia of the right ASC moved away from the ampulla after DHT and was cleared after the Yacovino maneuver (Supplementary Video 6).

#### 3.1.2 Mixed maneuver

Mixed maneuver: Step 1 was sitting position; In Step 2, all otoconia remained stationary but the head and body turned 90° to the right for preparation of the next step; In Step 3, the otoconia on the right HSC reached the outermost point and prepared to enter the nonampullary arm, at the same time, the right PSC and ASC otoconia moved toward the common crus; Step 4, the otoconia of the right HSC entered the nonampullary arm; Step 5, the right PSC and ASC otoconia moved further toward the common crus; Step 6, the right HSC otoconia in the nonampullary arm moved further toward the utricle; Step 7, the right PSC and ASC otoconia kept moving toward the common crus and the right HSC otoconia kept moving toward the utricle; Step 8, the right PSC and ASC otoconia moved toward the utricle and prepared to enter the common crus. In this step, the right HSC otoconia was cleared; In Step 9, the right PSC and ASC otoconia went through the common crus and returned the utricle (Supplementary Video 7; Figure 2).

### 3.2 Simulation of MM on BPPV VIEWER

High canalith burden test: As we activated the gravity, otoconia moved downward, the majority of otoconia located in the ampulla and its surroundings. Otoconia of ASC or PSC near the common crus and otoconia in nonampullary arm of HSC entered the utricle. Only the otoconia at the summit of ASC remained stationary. After the performance of MM, only a subset of otoconia were trapped in the ampulla of ASC, other otoconia returned into the utricle, then we performed MM again, and the otoconia trapped in the ampulla of ASC also repositioned into the utricle.

Movement trajectory of right side otoconia:

Step 1: Driven by gravity, the otoconia of ASC and HSC were trapped in the ampulla, whereas the otoconia of PSC moved away from the ampulla to the lowest position of PSC.

Step 2: All otoconia remained stationary.

Step 3: All otoconia moved away from the respective ampulla.

Step 4: Otoconia of ASC and HSC continued to move away from the ampulla, while otoconia of PSC migrated back toward the ampulla.

Step 5: Otoconia of HSC remained stationary; otoconia of PSC moved toward the common crus, and otoconia of ASC migrated away from the common crus.

Step 6: Otoconia of ASC and PSC moved toward the common crus, whereas otoconia of HSC migrated toward the utricle.

Step 7: Otoconia of ASC and PSC approached the common crus, but otoconia of PSC translocated to ASC, and otoconia of HSC entered the utricle.

Step 8: Otoconia originally from PSC (now in ASC) and otoconia of ASC otoconia both migrated away from the common crus in ASC; meanwhile, the otoconia that had entered the utricle moved upward but remain in the utricle.

Step 9: All otoconia were repositioned into the utricle.

## 4 Discussion

### 4.1 Servo motors platform and aVOR

The aVOR application is an excellent BPPV simulator that can be used to observe the impact of different head changes on otoconia. However, it can only simulate canalithiasis. To control head movements more accurately, we used SMP to quantitatively control head positional changes. In this study, the rotation angle of SM was controlled using a control board. The assembly of the SMP in this study was one-scheduled, and researchers could adjust the assembly scheme according to their needs. Different SMP assembly methods can affect the parameters of the rotating shafts. To avoid insufficient torque in the SM, an iPhone5 weighing 112 g was used. Although the SM was able to control the rotation angle, some errors may have occurred due to the mechanical structure. However, the CRMs were usually changed to 15°, 30°, 45°, 90°, and 120°, and the error in the SM rotation did not exceed ±1°, which did not affect the results. The semicircular canal angle in the aVOR app is theoretical, the orientation of the canals, ampulla, and other parts of the membranous labyrinth cannot be directly predicted and actual patients have many anatomical variations and pathological heterogeneity (20). Another limitation of the AVOR app is that only one clot of otoconia can be placed in each SC, precluding the observation of potential interactions between multiple otoconia.

### 4.2 BPPV VIEWER

To address the inability of the aVOR app to place multiple otoconia within a single semicircular canal, and to circumvent the limitations of single-model simulations, we utilized an additional downloadable software to verify the feasibility of the MM. A key advantage of this software is its capacity to place multiple otoconia within the same SC. We therefore placed the maximum number of otoconia achievable in the SCs of the model. Driven by gravity, some otoconia returned into the utricle, with the remainder predominantly accumulating in and around the ampullae. Following MM performance, only a subset of otoconia of ASC became trapped in the ampulla, while the rest were successfully repositioned into the utricle. The MM was thus repeated, and the remaining trapped otoconia were also repositioned into the utricle. Notably, the otoconia that had been repositioned into the utricle did not re-enter the SCs after the second performance of MM.

### 4.3 Classical CRMs

Currently, commonly used CRMs target a single semicircular canal, and posterior canal BPPV is the most common type of canalithiasis; therefore, the Epley maneuver is the most commonly used CRM. The initial Epley maneuver included an oscillator (12), however, it was subsequently found that the oscillator could not improve the curative effect (21). Hence, the Epley maneuver commonly used is a modified version. The result in this study showed that the Epley maneuver played a positive role in repositioning the otoconia of PSC, HSC and ASC, but only PSC otoconia returned into the utricle at last. Consequently, it is necessary to adopt further steps for repositioning the otoconia of HSC and ASC. In addition, inspired by the multifunctional effects of the Epley maneuver, we proposed the MM which could simultaneously treat otoconia in all three semicircular canals on one side.

Barbecue maneuver is another CRM frequently employed, which is useful for horizontal canal BPPV. Two types of Barbecue maneuvers are as follows: (1) turning 270° to the healthy side; and (2) turning 90° to the affected side and then 360° to the healthy side. By comparison, the head turned 90° to the affected side, which induced the otoconia to enter the nonampullary arm, resulting in a better outcome (22). So, the second type of Barbecue maneuver was selected in this study and the simulation of the Barbecue maneuver was in accordance with the existing literatures. Position of Step 3 and Step 4 in the Barbecue maneuver played a positive role in repositioning the otoconia on the same side, we adopted the positions as part of the mixed maneuver. Similarly, some positions of Gufoni maneuver and Yacovino maneuver were adopted.

The Gufoni maneuver is aimed at the HSC-BPPV with geotropic nystagmus (23), that is, the nonampullary arm otoconia in HSC. The ampullary arm otoconia of the HSC cannot be cleared by Gufoni maneuver, especially the otoconia trapped in the ampulla. In this study, we found that SRT can promote HSC otoconia to enter the nonampullary arm, facilitating the function of Gufoni maneuver on the HSC otoconia. As a diagnostic test, SRT was frequently used before the CRM, so Gufoni maneuver is clinically effective (24, 25).

Strangely, the Yacovino maneuver in isolation could clear no otoconia in the SMP-aVOR model, which does not match our clinical experience, and also differs from reports in the literature. Interestingly, after DHT, the Yacovino maneuver could clear the otoconia of the ASC. We found that in the initial state of this model, the ASC otoconia were trapped in the ampulla, after DHT, the otoconia moved away from the ampulla and entered the canal (26), then the Yacovino maneuver promoted the otoconia into the utricle. Anatomical variations may render the Yacovino maneuver ineffective. This is because a portion of otoconia in the ASC tends to become trapped in the ampulla under the influence of gravity in the upright position. If a sufficient angle is not achieved during the Yacovino maneuver to dislodge the otoconia from the ampulla, repositioning will be difficult to accomplish. As the most frequently used diagnostic test, DHT was usually performed before the CRM. DHT can facilitate the dislodgment of otoconia from the ampulla, thereby aiding in the repositioning of otoconia during the Yacovino maneuver. However, if the upright position is maintained for a sufficiently long period after DHT, the otoconia may become trapped in the ampulla once again. To demonstrate the potential role of DHT in the Yacovino maneuver, we shortened the interval between DHT and Yacovino maneuver.

In this study, the movement interval was 10 s to facilitate the observation of the effect based on the simplifications in the aVOR app. In an actual operation, due to more complex factors such as the density of the endolymph, the particle size of the canalith and other variables, the interval should exceed 45 s (27) or wait for the vertigo sensation to disappear or nystagmus to ensure that the otoconia movement is in place.

### 4.4 Mixed maneuver

The purpose of CRMs is to guide the otoconia back to the utricle via gravity or inertia. The design of the maneuver should consider the limitations of joint rotation in the human body. Otoconia stimulation was greatest when the semicircular canal was perpendicular to the ground. By combining the key positions of Classical CRMs, the otoconia of the three SCs on the same side can be cleared simultaneously. The MM can reduce the failure of otoconia clearance caused by an incorrect choice of maneuver or step. Because MM works on the three semicircular canal otoconia on the same side simultaneously, the diagnosis is mainly aimed at identifying which side is affected instead of which canal is affected. Observation of nystagmus elicited on positional tests is the gold standard for diagnosing the type of BPPV and deciding which canal is affected, but sometimes nystagmus is weak or momentary. In some cases, the process of positional test causing the displacement of otoconia may also complicate the diagnosis (8). A questionnaire independent of nystagmus was developed for self-diagnosis of BPPV (9), in which the first three questions were used to identify BPPV, question 4 was used to locate SCs, question 5 was used to identify the side, and question 6 was used to classify canalithiasis and cupulolithiasis. Since the questionnaire does not take nystagmus into account, it can only serve as a screening tool. However, we hope that the questionnaire or other screening tools combined with MM could facilitate the self-diagnosis and treatment of BPPV, which may reduce the discomfort caused by the positional test, incorrect recognition of nystagmus, failure caused by the inappropriate selection of CRMs or incorrect operation steps, and possible risk of canal switch (28). We have found that MM also poses a risk of causing canal switch. However, the otoconia of the three SCs can eventually be repositioned. If excessive otoconia cause canalith jam, performing MM again could lead to further success.

The final step of the MM is the sitting position, but the angle of head-down rotation may influence the probability of treatment failure. To avoid a high probability of treatment failure, 45°–75° head-down position should be maintained in the final step (29). Therefore, a 45-degree head-down position in the final step of the MM should be adopted as a mandatory safety precaution.

### 4.5 Limitations

We intended to reposition all types of otoconia with a single CRM. However, as the MM maneuver is merely a combination of classic CRMs, it fails to achieve effective repositioning for otoconia that are refractory to classic CRMs, such as cupulolithiasis and canalolithiasis involving the short arm of PSC. Since neither of the two simulation models strictly distinguished the anatomical structures of the utricle and the short arm of PSC, this subset of cases was not included in our results. Although bow-and-yaw maneuver has been proposed as a convenient and comfortable manner for the short-arm type PSC BPPV (30), maintaining a 45-degree head-down position in the final step of the MM is critical to prevent otoconia from entering the short arm of PSC. In the future, the MM maneuver may not be limited to classic CRMs, but instead incorporate more CRMs, such as bow-and-yaw maneuver, Zuma maneuver (16), Gans maneuver (31) and other variations or modifications of classic CRMs. As canalolithiasis is the most common subtype of BPPV, free-floating otoconia are frequently located in the long arm of PSC, the ampullary and nonampullary arm of HSC, and the ampullae of ASC and HSC. Our simulation results have demonstrated the efficacy of the MM for repositioning these otoconia.

The bilateral SCs that lie in a common plane constitute functional pairs. Although this study focused on the efficacy of the MM in repositioning otoconia in the three SCs on the same side, potential contralateral effects should not be overlooked. Performing the MM on one side may theoretically induce potential otoconia to migrate into the contralateral SCs. This iatrogenic complication is clinically relevant and should not be neglected. Careful clinical follow-up after repositioning therapy is also of great importance.

We have only verified the efficacy of the MM from a geometric perspective using two models. Given the anatomical complexity of SCs, more models based on real human anatomical data will be required in future research, and rigorous clinical trials need to be conducted to validate the clinical effectiveness of the MM and further optimize its procedures.

Step 2 of the MM only involves body rotation, with no effective stimulation exerted on otoconia repositioning. This is because the rotational chairs commonly used in clinical practice are biaxial devices (7); the same steps of the MM are adopted for both manual and mechanical performance to facilitate its application in future research. Given that the MM includes more steps than the classical CRMs, it is not consistent with the Minimum Stimulus Strategy (32) and prolongs the treatment duration. Therefore, classical CRMs should still be the first-line option when the otoconia location is clearly identified. The MM is not designed to replace existing CRMs, but rather to serve as an additional optional strategy for clinical practice.

## 5 Conclusion

The SMP-aVOR model and BPPV VIEWER provide methods for studying otoconia repositioning. The MM hybridized by classical CRMs showed potential capability to reposition the common types of otoconia in the three SCs on the same side. However, further research and investigation are required for its application in special subtypes. The MM provides a novel therapeutic strategy, and its efficacy and safety need to be further verified in clinical practice.

## Data Availability

The original contributions presented in the study are included in the article/Supplementary material, further inquiries can be directed to the corresponding author.

## Supplementary material

SUPPLEMENTARY VIDEO 1 Simulation of Epley maneuver.

SUPPLEMENTARY VIDEO 2 Simulation of Barbecue maneuver.

SUPPLEMENTARY VIDEO 3 Simulation of Gufoni maneuver.

SUPPLEMENTARY VIDEO 4 Simulation of SRT + Gufoni maneuver.

SUPPLEMENTARY VIDEO 5 Simulation of Yacovino maneuver.

SUPPLEMENTARY VIDEO 6 Simulation of DHT + Yacovino maneuver.

SUPPLEMENTARY VIDEO 7 Simulation of mixed maneuver.
