# Supplementary material for: Three-dimensional simulations of mixed maneuver for three semicircular canalithiasis on the same side
Source: Front Med (Lausanne). 2026 Mar 13;13:1782263. doi: 10.3389/fmed.2026.1782263 (PMC13021767; doi:10.3389/fmed.2026.1782263)
Supplement: Supplementary file 8 [file Table_1.docx]

|  | **EM** | | | | | | **BM** | | | | | | **GM** | | | | | | | **SRT+GM** | | | | | | | **YM** | | | | | | | **DHT+YM** | | | | | | | **MM** | | | | | | |  |
| --- | --- | --- | --- | --- | --- | --- | --- | --- | --- | --- | --- | --- | --- | --- | --- | --- | --- | --- | --- | --- | --- | --- | --- | --- | --- | --- | --- | --- | --- | --- | --- | --- | --- | --- | --- | --- | --- | --- | --- | --- | --- | --- | --- | --- | --- | --- | --- | --- |
|  | LP | LH | LA | RP | RH | RA | LP | LH | LA | RP | RH | RA | | LP | LH | LA | RP | RH | RA | | LP | LH | LA | RP | RH | RA | | LP | LH | LA | RP | RH | RA | | LP | LH | LA | RP | RH | RA | | LP | LH | LA | RP | RH | RA | |
| **S1** | - | - | - | - | - | - | - | - | - | - | - | - | | - | - | - | - | - | - | | - | - | - | - | - | - | | - | - | - | - | - | - | | - | - | - | - | - | - | | - | - | - | - | - | - | |
| **S2** | - | - | - | - | - | - | u | - | - | u | - | - | | u | u | u | a | - | - | | u | - | - | u | - | - | | u | - | - | u | - | - | | - | - | - | - | - | - | | - | - | - | - | - | - | |
| **S3** | - | - | - | u | u | u | - | - | - | - | u | u | | - | - | - | - | - | - | | - | - | - | - | u | u | | a | - | - | a | - | - | | - | - | - | u | u | u | | a | - | a | u | u | u | |
| **S4** | u | u | u | u | - | u | - | - | - | - | u | - | | a | - | a | u | - | - | | - | - | - | - | u | - | | a | - | - | a | - | - | | - | - | - | a | - | a | | - | - | - | u | u | - | |
| **S5** | - | - | a | u | a | u | - | u | u | - | u | - | |  |  |  |  |  |  | | a | - | - | a | - | a | |  |  |  |  |  |  | | u | - | - | u | - | u | | - | - | - | - | u | u | |
| **S6** | a | - | a | u | - | u | - | a | - | - | u | - | |  |  |  |  |  |  | | u | u | u | a | u | - | |  |  |  |  |  |  | | a | - | - | a | u | u | | - | u | u | u | u | u | |
| **S7** |  | | | | | | - | a | - | - | u | - | |  |  |  |  |  |  | | - | a | - | - | u | - | |  |  |  |  |  |  | | a | - | - | a | - | u | | - | u | - | u | u | u | |
| **S8** |  |  |  |  |  |  | a | - | a | a | - | a | |  |  |  |  |  |  | | - | - | a | u | - | - | |  |  |  |  |  |  | |  |  |  |  |  |  | | - | a | a | u | u | - | |
| **S9** |  |  |  |  |  |  |  |  |  |  |  |  | |  |  |  |  |  |  | |  |  |  |  |  |  | |  |  |  |  |  |  | |  |  |  |  |  |  | | u | - | a | u | - | u | |

Table 1. The effects on otoconia in each step of CRMs for right canal. “u” indicates that otoconia move toward the utricle; “a” indicates that otoconia move toward the ampulla, “-” indicates that otoconia remain stationary. S1~S9: Step1~Step9 of each CRMs; EM: Epley maneuver; BM: Barbecue maneuver; GM: Gufoni maneuver; SRT + GM: Supine roll test + Gufoni maneuver; YM: Yacovino maneuver; DHT + YM: Dix-Hallpike test + Yacovino maneuver; MM: Mixed maneuver; LP: otoconia of left posterior semicircular canal; LH: otoconia of left horizontal semicircular canal; LA: otoconia of left anterior semicircular canal; RP: otoconia of right posterior semicircular canal; RH: otoconia of right horizontal semicircular canal; RA: otoconia of right anterior semicircular canal
